# Clinical Trials Corner: September 2017

**DOI:** 10.3233/JHD-170262

**Published:** 2017-09-29

**Authors:** Filipe B. Rodrigues, Edward J. Wild

**Affiliations:** a Huntington’s Disease Centre, University College London, London, UK; b Laboratory of Clinical Pharmacology and Therapeutics, Faculty of Medicine, University of Lisbon, Lisbon, Portugal; c Clinical Pharmacology Unit, Instituto de Medicina Molecular, Lisbon, Portugal

**Keywords:** Clinical trials, Huntington’s disease

## Abstract

Clinical Trials Corner of Journal of Huntington’s Disease will regularly review ongoing and recently completed clinical trials in Huntington’s disease. In this inaugural issue, we list all currently registered and ongoing clinical trials, expand on LEGATO-HD and IONIS-HTT_Rx_, and cover two recently finished trials: Amaryllis and Pride-HD.

## INTRODUCTION

Clinical Trials Corner of *Journal of Huntington*’*s Disease* is a new, regular, peer-reviewed section devoted to highlighting ongoing or recently completed clinical trials in Huntington’s disease (HD).

To do so, we will gather and curate data from the World Health Organization (WHO) International Clinical Trials Search Portal (ICTRP)— a central database that contains the trial registration datasets provided by 17 clinical trial registries [1], including the EU Clinical Trials Register (EU-CTR), the USA ClinicalTrials.gov, among others— using the keywords “*Huntington*’*s*” and “*Huntington*”. As trial registration has been settled as a condition for publication by the International Committee of Medical Journal Editors (ICMJE) since 2005 [2], and the following year the WHO supported this measure, this strategy is expected to harvest the majority of ongoing clinical trials [3]. We will use only publicly available information to describe the trials and theirresults.

There are only two drugs specifically approved for HD [4]: tetrabenazine [5] and deutetrabenazine [6], both with a moderate effect on involuntary movements. No intervention has shown to modify disease progression so far [7]. That being said, almost one hundred clinical trials and 50 different interventions have been or are currently being tested in HD [8]. It is clear that modifying the progression of HD is exceptionally difficult; that success in preclinical models so far has failed to anticipate the outcome of subsequent human trials; and that there is a need for not only better drugs, but better means of deciding which drugs we should test in patients.

In this inaugural Clinical Trials Corner, we will list all currently registered and ongoing clinical trials, expand on LEGATO-HD (NCT02215616) and IONIS-HTT_Rx_ (NCT02519036), and cover two recently finished trials: Amaryllis (NCT02197130) and Pride-HD (NCT02006472). For future editions, we will summarize current efforts and recent developments as well as providing in-depth information on notable trials.

If you would like to draw attention to specific trials, please feel free to email us at: f.rodrigues@ucl.ac.uk; e.wild@ucl.ac.uk.

## ONGOING CLINICAL TRIALS

A list of ongoing clinical trials is given in Tables 1, 2 and 3.

**Table 1:** Ongoing pharmacological clinical trials registered at the World Health Organization (WHO) International Clinical Trials Research Platform (ICTRP) for people with Huntington’s disease (HD). NINDS, National Institute of Neurological Disorders and Stroke; HSG, Huntington Study Group; N/S, not specified; PD, Parkinson’s disease; VMAT2, Vesicular Monoamine Transporter 2

Registration ID	Trial name	Intervention	Mechanism of	Population	Comparison	Main outcome	Study design	Estimated	Sponsor	Location
			Action					Enrolment
**EUCTR2016-003730-25-NL**	CHALLENGE-HD	SBT-020	Mitochondria-targeted cytoprotective peptide	Early HD	Placebo	Safety and tolerability at 7 and 28 days	Randomized, double-blind, placebo-controlled, parallel trial	24	Stealth Biotherapeutics	Netherlands (single center)
**NCT03019289**	–	Pridopidine	Dopaminergic stabilizer	Healthy individuals and HD	–	Pharmacodynamic at 1 day	Single dose, open-label, single group trial	38	Teva Branded Pharmaceutical Products, R&D Inc.	Germany (single center)
**NCT02453061**	TRIHEP 3	Triheptanoin	Anaplerotic therapy	HD	Placebo	Pharmacodynamic efficacy at 6 months	Randomized, double-blind, placebo-controlled, parallel trial	100	Institut National de la Santé Et de la Recherche Médicale, Ultragenyx Pharmaceutical Inc.	France, Netherlands (multi center)
**NCT02519036**	IONIS-HTT_RX_	IONIS-HTT_RX_	Antisense oligonucleotide	HD	Placebo	Safety and tolerability at 29 weeks	Randomized, double-blind, placebo-controlled, parallel, dose ascending trial	46	Ionis Pharmaceuticals, Inc.	Canada, Germany, UK (multi center)
**NCT02509793**	–	Tetrabenazine	VMAT2 inhibitor	HD with impulsivity	–	Cognitive and behavioral effects at 8 weeks	Single group, open-label trial	20	University of Texas Health Science Center, and H. Lundbeck A/S	USA (single center)
**NCT02507284**	STAIR	SRX246	Vasopressin 1a Receptor Antagonist	Early and moderate HD with irritability	Placebo	Feasibility at 12 weeks	Randomized, double-blind, placebo-controlled, parallel trials	108	Azevan Pharmaceuticals, NINDS, & NeuroNEXT Network	USA (multi center)
**NCT02494778**	Open PRIDE-HD	Pridopidine	Dopaminergic stabilizer	PRIDE-HD completers	–	Safety at 104 weeks	Single group, open label extension of PRIDE-HD	300	Teva Branded Pharmaceutical Products, R&D Inc.	Australia, Austria, Canada, Denmark, France, Germany, Italy, Netherlands, Poland, Russia, UK, USA (multi center)
**NCT02481674**	SIGNAL	VX15/2503	Anti-semaphorin 4D monoclonal antibody	Late premanifest or early HD	Placebo	Safety and tolerability at 15 and 21 months	Randomized, double-blind, placebo-controlled, parallel trial	116	Vaccinex Inc., HSG	USA (multi center)
**NCT02336633**	REVHD	Resveratrol	Dietary supplement	HD	Placebo	Neuroimaging biomarkers at 1 year	Randomized, double-blind, placebo-controlled, parallel trial	102	Assistance Publique –Hôpitaux de Paris	France (multi center)
**NCT02215616**	LEGATO-HD	Laquinimod	Immunomodulatory molecule	HD	Placebo	Efficacy at 1, 3, 6, and 12 months	Randomized, double-blind, placebo-controlled, parallel trial	400	Teva Branded Pharmaceutical Products, R&D Inc.	Canada, Czech Republic, France, Germany, India, Israel, Italy, Netherlands, Portugal, Russia, Spain, UK, USA (multi center)
**EUCTR2013-002545-10-SE**	OSU6162Open1309	(–)-OSU616	Monoaminergic stabilizer	HD, PD, brain trauma, stroke, myalgic encephalomyelitis and narcolepsy	–	Safety at 3, 6 and 12 months	Single group, open-label trial	240	A. Carlsson Research AB	Sweden (multi center)
**NCT00652457**	MEM-HD	Memantine	NMDA receptor antagonist	HD and memory or concentration difficulties	Placebo	Efficacy at 3 and 6 months	Randomized, double-blind, placebo-controlled, cross-over trial	60	University of California, San Diego, Forest Laboratories	USA (multi center)
**NCT00632645**	NEUROHD	Olanzapine	Dopamine agonist	HD with motor or behavioral symptoms	Tetrabenazine, or tiapride	Efficacy at 12 months	Randomized, open-label, controlled, parallel trial	180	Assistance Publique –Hôpitaux de Paris,	France (single center)
**NCT01306929**	OPEN-HART	Pridopidine	Dopaminergic stabilizer	HART or PRIDE-HD completers	–	Safety at 2 years	Single group, open label extension of HART	235	Teva Branded Pharmaceutical Products, R&D Inc.	Canada, USA (multi center)
**NCT00514774**	UDCA-HD	Ursodiol	Bile acid	HD	Placebo	Safety, tolerability and pharmacokinetics at 35 days	Randomized, double-blind, placebo-controlled, parallel trial	21	Oregon Health and Science University, HSG, Huntington Society of Canada	N/S
**NCT01897896**	ARC-HD	Deutetrabenazine	VMAT2 inhibitor	Early and moderate HD with chorea on tetrabenazine or FIRST-HD completers	–	Safety at 54 weeks	Single group, open-label, drug-switching trial	238	Auspex Pharmaceuticals, Inc., Teva Pharmaceutical Industries	Australia, Canada, USA (multi center)
**ACTRN12616001611415**	VCAS-HD	Varenicline	Nicotinic acid receptor partial agonist	HD	Placebo	Efficacy at 10 weeks	Randomized, double-blind, placebo-controlled, parallel trial	40	University of Auckland	New Zealand (single center)

**Table 2:** Ongoing invasive non-pharmacological clinical trials registered at the World Health Organization (WHO) International Clinical Trials Research Platform (ICTRP) for people with Huntington’s disease (HD). DBS, deep brain stimulation; EHDN, European Huntington’s Disease Network; ET, Essential Tremor; GP, Globus pallidus; HT, Holmes Tremor; MNC, mononuclear cells; PD, Parkinson’s disease; TD, Tardive dyskinesia; WD, Wilson’s disease

Registration ID	Trial name	Intervention	Mechanism	Population	Comparison	Main outcome	Study design	Estimated	Sponsor	Location
			of Action					Enrolment
**NCT02535884**	HD-DBS	GP DBS	Deep brain stimulation	Moderate HD with chorea	Sham intervention	Efficacy at 12 months	Randomized, double-blind, sham-controlled, parallel trial	50	Heinrich-Heine University, KKS Netzwerk, Medtronic, The George Institute, EHDN, CHDI Foundation, Inc.	Austria, Germany, Switzerland (multi center)
**NCT01834053**	BMACHC	Bone Marrow Derived MNC transplant	Bone marrow transplant	HD with chorea	–	Cognitive and behavioral effects at 6 months	Single group, open-label trial	50	Chaitanya Hospital, Pune	India (single center)
**NCT02263430**	–	GP DBS	Deep brain stimulation	HD with chorea	Sham stimulation	Efficacy at 12 months	Randomized, double-blind, placebo-controlled, parallel trial	8	Beijing Pins Medical Co., Ltd, Beijing Tiantan Hospital	China (single center)
**NCT02252380**	–	Magnetic Resonance Guided Focused Ultrasound	Extracranial stereotactic radioablation	HD, ET, HT, PD, WD, dystonia, TD, or orofacial dyskinesias	–	Adverse events after the procedure	Single group, open-label trial	10	InSightec	Canada (single center)

**Table 3:** Ongoing non-invasive non-pharmacological clinical trials registered at the World Health Organization (WHO) International Clinical Trials Research Platform (ICTRP) for people with Huntington’s disease (HD). AD, Alzheimer’s disease; ALS, Amyotrophic Lateral Sclerosis; ET, Essential Tremor; HT, Holmes Tremor; MS, Multiple Sclerosis; PD, Parkinson’s disease; TD, Tardive dyskinesia

Registration ID	Trial name	Intervention	Mechanism of	Population	Comparison	Main outcome	Study design	Estimated	Sponsor	Location
			Action					Enrolment
**NCT02990676**	CogTrainHD	Computerized Cognitive Training	Cognitive training	HD	No intervention	Feasibility at 4 years	Open-label, controlled, parallel trial	50	Cardiff University	UK (single center)
**NCT01879267**	–	Endurance exercise training	Physiotherapy	HD and healthy controls	–	Motor effects 6 months	Single group, open-label trial with parallel healthy controls arm	40	University of Zurich	Switzerland (single center)
**NCT02464293**	–	Mindfulness-based Cognitive Therapy	Cognitive therapy	Premanifest and early HD with behavioral symptoms	–	Behavioral effect at 2 weeks, 3 months and 1 year	Single group, open-label trial	16	Lancaster University, Central Manchester University Hospitals NHS Foundation Trust	UK (single center)
**NCT02216474**	–	tDCS	Transcranial magnetic stimulation	HD or Tourette Syndrome	Sham stimulation	Efficacy at 2 weeks	Randomized, double-blind, placebo-controlled, cross-over trial	100	Birmingham and Solihull Mental Health NHS Foundation Trust, University of Birmingham	UK (single center)
**NCT02750982**	–	Laughter Therapy	Cognitive therapy	HD, AD, ALS, brain injury, MS, PD, post/stroke or spinal cord injury	–	Behavioral effects at 8 weeks	Single group, open-label trial	24	Brown, Theodore R., M.D., MPH	USA (single center)
**NCT01602276**	–	tDCS	Transcranial magnetic stimulation	Subcortical brain damage, including HD	Sham stimulation	Efficacy at 1 month	Randomized, single-blind, placebo-controlled, cross-over trial, with parallel healthy control arm	150	Johns Hopkins University	USA (single center)

### LEGATO-HD (NCT02215616)

*Study title:* A Clinical Study in Subjects With Huntington’s Disease to Assess the Efficacy and Safety of Three Oral Doses of Laquinimod [9].

*Intervention:* Laquinimod, an immunomodulatory molecule [10].

*Description:* The LEGATO-HD trial aims to compare the efficacy and safety of laquinimod 0.5 mg qd, 1 mg qd, 1.5 mg qd, and placebo qd, for disease modification in people with HD (CAG repeat number ≥ 36 plus Unified Huntington’s Disease Rating Scale (UHDRS) Total Motor Score (TMS) >5), aged between 21 and 55 years old.

Participant involvement will last for 12 months of treatment. The trial is a phase 2, international, multi-center, randomized, placebo controlled, double blind, parallel study. The recruitment aim is 400 participants in Canada, Czech Republic, France, Germany, India, Israel, Italy, Netherlands, Portugal, Russia, Spain, United Kingdom, and United States of America.

The primary outcome is change from baseline in the UHDRS TMS after 1, 3, 6, and 12 months of treatment. The secondary outcomes involve the UHDRS Total Functional Capacity (TFC), the Clinician’s Interview-Based Impression of Change Plus Caregiver Input (CIBIC-Plus) global score, the Huntington’s Disease Cognitive Assessment Battery (HD-CAB), and caudate volume.

*Sponsors/funders:* Teva Branded Pharmaceutical Products, R&D Inc.

*Comments:* Along the course of this study, due to safety concerns derived from a study of laquinimod in multiple sclerosis, the sponsor opted to stop the 1.5 mg qd dosage but maintain the others. This study is now fully recruited [11] with an expected completion date of August 2018.

### IONIS-HTT_Rx_ (NCT02519036)

*Study title:* Safety, Tolerability, Pharmacokinetics, and Pharmacodynamics of IONIS-HTT_Rx_ in Patients With Early Manifest Huntington’s Disease [12]

*Intervention:* IONIS-HTT_Rx_, an antisense oligonucleotide against the huntingtin pre-messenger RNA [13].

*Description:* The IONIS-HTT_Rx_ trial aims to assess the safety, tolerability, pharmacokinetics and pharmacodynamics of multiple ascending doses of IONIS-HTT_Rx_ administered intrathecally, comparing with intrathecal placebo, for disease modification in people with HD, aged between 25 and 65 years old. HTT_Rx_ is an antisense oligonucleotide targeting the pre-mRNA transcript of the HTT gene in an allele-nonselective manner, with the aim of reducing the production of mutant huntingtin protein. The intervention is administered 4 times at 4 week intervals over the course of 13 weeks. The dose has ascended consecutively throughout the study.

Each participant’s involvement will last for 29 weeks. It is a phase 1b/2a, international multi-center, randomized, placebo controlled, double blind, parallel, dose-ascending study, taking place in Canada, Germany and the United Kingdom. The recruitment goal of 46 participants was reached in June2017 [14].

The primary outcome is safety and tolerability at 29 weeks. The secondary outcomes involve pharmacokinetic and pharmacodynamic measures in the cerebrospinal fluid, such as peak drug concentrations, time to peak dose concentrations, huntingtin concentration, neurofilament light concentration, and also ventricular volume and performance on the HD Cognitive Assessment Battery (HD-CAB).

*Sponsors/funders:* Ionis Pharmaceuticals, Inc.

*Comments:* This trial is currently fully recruited and an open-label extension was announced in June 2017 for the participants in the original trial.

## COMPLETED CLINICAL TRIALS

### Amaryllis (NCT02197130)

*Study title:* Randomized, Placebo Controlled Study Of The Efficacy And Safety Of PF-02545920 In Subjects With Huntington’s Disease [15].

*Intervention:* PF-02545920, a phosphodiesterase 10a inhibitor [16].

*Description:* The goal of the Amaryllis trial was to compare the efficacy and safety of PF-02545920 5 mg bid, PF-02545920 20 mg bid, and placebo bid, for symptomatic relief of motor impairment in people with early HD (CAG repeat number ≥ 36 plus UHDRS TFC ≥ 7) and chorea (UHDRS TMS ≥ 10), aged between 30 and 65 years old.

Participant involvement lasted for 26 weeks. It was a phase 2, international, multi-center, randomized, placebo controlled, double blind, parallel study conducted in Canada, Germany, Poland, United Kingdom, and United States of America. 272 participants were recruited.

The primary outcome was change from baseline in the UHDRS TMS after 26 weeks of treatment. The secondary outcomes involved the UHDRS TFC, the Clinical Global Impression-Improvement, the Columbia Suicide Severity Rating Scale (C-SSRS), extrapyramidal symptoms, and white cell counts and neutrophil counts.

*Sponsors/funders:* Pfizer

*Results:* The trial was completed on September 2016. Although the results have not been released in a peer-reviewed publication, Pfizer has officially announced that the phosphodiesterase 10a inhibitor did not meet its goals in improving motor impairment in people with HD. Indeed several other secondary outcomes remained unchanged, such as functional ability [17]. As a consequence, the ongoing 12 month-long open-label extension (NCT02342548) of PF-02545920 20 mg bid was discontinued in February 2017 [18].

### Pride-HD (NCT02006472)

*Study title:* A Phase 2, to Evaluating the Safety and Efficacy of Pridopidine Versus Placebo for Symptomatic Treatment in Patients With Huntington’s Disease [19].

*Intervention:* Pridopidine, a dopaminergic stabilizer [20].

*Description:* Pride-HD trial aimed to compare the efficacy and safety of pridopidine 45 mg bid, 67.5 mg bid, 90 mg bid, 112.5 mg bid, and placebo bid, for symptomatic relief of motor impairment in people with HD (CAG repeat number ≥ 36 plus UHDRS Independence Score <90%) and chorea (UHDRS TMS ≥ 25), aged ≥ 21 years old.

It lasted for 52 weeks, was a phase 2, international, multi-center, randomized, placebo controlled, double blind, parallel study taking place in Australia, Austria, Canada, Czech Republic, Denmark, France, Germany, Italy, Netherlands, Poland, Russia, United Kingdom, and United States of America. It recruited 408 participants.

The primary outcome was change from baseline in the UHDRS TMS after 26 weeks of treatment. The secondary outcomes involved the modified Physical Performance Test, and adverse events. Early in the course of the study, the sponsors instituted a change in the study design, from a 26-week study focused primarily on changes in motor symptoms as measured by the TMS, to a longer 52-week study to explore pridopidine’s potential impact on functionalendpoints.

*Sponsors/funders:* Teva Branded Pharmaceutical Products, R&D Inc., European Huntington’s Disease Network (EHDN) and Huntington’s Study Group (HSG).

*Results:* The trial was completed on July 2016. On September 2016 Teva announced on its website that “*pridopidine demonstrates slowing of progression of Huntington disease in Pride-HD study as measured by total functional capacity*” [21]. This announcement sparked some controversy [22], since the data presented by Teva at the 9th EHDN Plenary Meeting 2016 The Hague showed that Pride-HD failed to meet its primary endpoint –change in UHDRS TMS at 26 weeks [21]. The interpretation of potential benefit hinged upon of a lack of decline in the UHDRS TFC at the extended 52-week timepoint. The effect was only significant in patients taking the lowest dose of pridopidine. The sponsor also pointed out “*an unusually high placebo effect*” that complicated interpretation of the findings. The Chairs of the EHDN Executive Committee issued a statement responding to Teva’s announcement, saying “*there has been discussion over the statement in the press release that these results indicate that pridopidine slows down disease progression in HD. This statement needs to be read in the context of the whole document, which clearly speaks about slowing decline in functional capacity. This should not be misunderstood as a demonstration of disease modification or ofneuroprotection*” [23].

Still, the final results of this study have not yet been published, and we look forward to seeing them after peer review. The open label extension of this trial— Open PRIDE-HD (NCT02494778)— is still ongoing, according to the latest public information.

## CONFLICTS OF INTEREST

FBR and EJW are sub-investigators on LEGATO-HD (NCT02215616) and IONIS-HTT_Rx_ (NCT02519036), and EJW was a sub-investigator on Amaryllis (NCT02197130). The authors did not make use of confidential or privileged information: all materials included in this manuscript were collected from publicly available sources. EJW has participated in scientific advisory boards with Hoffmann-La Roche Ltd, Ionis, Shire, GSK and Wave Life Sciences. All honoraria were paid through UCL Consultants Ltd, a wholly owned subsidiary of UCL. Their Host Institution, University College London Hospitals NHS Foundation Trust, has received funds as compensation for conducting clinical trials for Ionis Pharmaceuticals, Pfizer and Teva Pharmaceuticals.
